# SAG/RBX2 E3 Ubiquitin Ligase Differentially Regulates Inflammatory Responses of Myeloid Cell Subsets

**DOI:** 10.3389/fimmu.2018.02882

**Published:** 2018-12-06

**Authors:** Xiufang Xiong, Nathan D. Mathewson, Hua Li, Mingjia Tan, Hideaki Fujiwara, Haomin Li, Pavan Reddy, Yi Sun

**Affiliations:** ^1^Institute of Translational Medicine, and Cancer Institute of the Second Affiliated Hospital, Zhejiang University School of Medicine, Hangzhou, China; ^2^Department of Radiation Oncology, University of Michigan, Ann Arbor, MI, United States; ^3^Division of Hematology and Oncology, Department of Internal Medicine, University of Michigan Comprehensive Cancer Center, Ann Arbor, MI, United States; ^4^Graduate Program in Immunology, University of Michigan Medical School, Ann Arbor, MI, United States; ^5^Department of Cancer Immunology and Virology, Department of Microbiology and Immunobiology, Department of Neurology, Dana-Farber Cancer Institute, Harvard Medical School, and Brigham and Women's Hospital, Boston, MA, United States; ^6^Children's Hospital, Zhejiang University School of Medicine, Hangzhou, China

**Keywords:** inflammatory response, macrophage, neutrophil, SAG ubiquitin ligase, LPS

## Abstract

Macrophages form an important component of the innate immune system and serve as first responders against invading pathogens. While pathways critical for initiation of inflammatory responses between macrophages and other LysM^+^ myeloid cells are largely similar, it remains unknown whether a specific pathway has differential effects on inflammatory responses mediated between these cells. Recent studies demonstrated that depletion of SAG (Sensitive to Apoptosis Gene), an E3 ubiquitin ligase, blocked inflammatory responses generated by macrophages and dendritic cells in response to LPS in cell culture settings. However, the *in vivo* role of Sag on modulation of macrophages and neutrophil is not known. Here we generated LysM-Cre/*Sag*^*fl*/*fl*^ mice with selective *Sag* deletion in myeloid lineage, and found that in contrast to *in vitro* observations, LysM-Cre/*Sag*^*fl*/*fl*^ mice showed increased serum levels of proinflammatory cytokines and enhanced mortality in response to LPS. Interestingly, while *Sag*^−/−^ macrophages released less proinflammatory cytokines, *Sag*^−/−^ neutrophils released more. Mechanistically, expression of a list of genes response to LPS was significantly altered in bone marrow cells from *LysM-Cre*^+^/*Sag*^*fl*/*fl*^ mice after LPS challenge. Specifically, induction by LPS of myeloperoxidase (Mpo), a key neutrophil enzyme, and Elane, neutrophil expressed elastase, was significantly decreased upon Sag depletion. Collectively, our study revealed that Sag plays a differential role in the activation of macrophages and neutrophils.

## Introduction

The innate immune system consists of several cell types and is the first line of defense in the host, functioning as a barrier to infection from invading organisms (1). Macrophages and neutrophils, two cell types of the innate immune system, derive from the myeloid progenitor lineage that are critical for the initiation of inflammation in the context of antimicrobial immunity (2). Innate immune cells recognize and bind to pathogen associated molecular patterns (PAMPs) of pathogens via pattern recognition receptors (PRRs). Toll-like receptors (TLRs), a type of PRR, regardless of the innate immune cells that express them, utilize similar signal pathways that lead to the activation of the nuclear factor-kappa B (NF-κB) pathway (3) and initiates the release of proinflammatory cytokines (4, 5). These mutual and complementary features of innate immune cells likely favor their interaction and cooperation in generating complimentary innate immune responses (2). However, whether there are differences in the mechanisms through which specific innate immune cells are activated in response to infection remains unknown. In this context, specifically, whether the critical LysM-enzyme expressing innate immune cells—namely macrophages and neutrophils—utilize the same pathway to produce differential inflammatory responses is not known.

SAG, also known as RING box protein 2 (RBX2), Regulator of Cullins 2 (ROC2), or RING Finger Protein 7 (RNF7)—was originally cloned by differential display in our laboratory (6, 7). As a member of the Cullin-RING ligase (CRL) E3 ubiquitin ligase complex, SAG binds to ubiquitin-loaded E2 and catalyzes the ubiquitin transfer from the E2 molecule to a substrate for subsequent degradation by the proteasome. By promoting the degradation of a variety of substrates through its ligase activity, SAG regulates diverse signaling pathways and biological processes, including cell apoptosis, embryonic development, vasculogenesis, angiogenesis, and tumorigenesis (8). SAG was previously reported to maintain macrophage survival and to regulate the levels of inflammatory cytokines in response to infection by ubiquitinating the pro-apoptotic proteins Bax and SARM (9, 10). Further, inhibitor of κB (IκBα), a classical inhibitor of NF-κB activation, is a direct substrate of the SAG-CRL1^β−*TrCP*^ E3 ubiquitin ligase (11). In addition, SAG is also one of several E3 ligases for neddylation, which is required for CRL activation by cullin neddylation (12). Accumulating studies have shown that neddylation pathway regulates Lipopolysaccharide (LPS)-induced proinflammatory cytokine production in macrophages by blocking CRL-mediated IκBα degradation to retain NF-κB in the cytoplasm (10, 13).

It is well-known that the activation and translocation of NF-κB to the nucleus is responsible for the transcription of the proinflammatory cytokines tumor necrosis factor-alpha (TNFα) and interleukin-6 (IL-6) (14, 15). The accumulation of IκB upon SAG disruption leads to the inhibition of NF-κB activation and thus decreases inflammatory responses by dendritic cells (DCs) *in vitro* (16). Thus, inhibition of SAG may regulate the innate immune response of macrophages in a similar manner as in DCs (16).

LysM^+^ cells (i.e., macrophages and neutrophils) are the keys to the *in vivo* inflammatory response. In light of the regulation of NF-κB by SAG, we tested the hypothesis that *Sag* deficient LysM^+^ cells would release less proinflammatory cytokines in response to LPS and mitigate LPS-induced mortality. Surprisingly, we found that loss of Sag protein in these cells resulted in a net increase in proinflammatory cytokines and furthermore increased LPS-induced mortality. Mechanistic studies showed that disruption of Sag in macrophages, is consistent with previous reports (9), reduced the release of inflammatory cytokines by preventing the degradation of IκBα and thus inhibited subsequent NF-κB activation. By contrast, *Sag* deficiency in neutrophils up-regulated the release of inflammatory cytokines with no change on nuclear translocation of NF-κB after LPS stimulation. Furthermore, microarray analysis revealed that induction of myeloperoxidase (Mpo), a key neutrophil enzyme (17), and Elane, neutrophil expressed elastase (18), was significantly decreased in bone marrow cells from *LysM-Cre*^+^/*Sag*^*fl*/*fl*^ mice in response to LPS, which likely contributed to the net increase in systemic levels of proinflammatory cytokines and increased LPS-induced mortality in response to LPS *in vivo*. Taken together, these findings suggest that the same molecule—namely Sag—plays a differential role in the activation of macrophages and neutrophils.

## Materials and Methods

### Reagents

RPMI, penicillin and streptomycin, and sodium pyruvate were purchased from Gibco (Grand Island, NY); FCS from GemCell (Sacramento, CA); 2-ME from Sigma (St. Louis, MO); murine GM-CSF from Peprotech (Rocky Hill, NJ). All antibodies (Abs) used for FACS were purchased from eBioscience (San Diego, CA). DMSO and lipopolysaccharide (LPS) was purchased from Sigma (St. Louis, MO).

### Mouse Studies

The *Sag*^*fl*/*fl*^ mice were generated as previously described (19, 20). The LysM-cre mice were purchased from the Jackson Laboratory (Stock number: 004781). Male and female mice used were between 8 and 12 weeks of age. All procedures were approved by the University of Michigan Committee on Use and Care of Animals. Animal care was provided in accordance with the principles and procedures outlined in the National Research Council Guide for the Care and Use of Laboratory Animals.

### LPS Administration

Mice at 8 weeks old were subjected to i.p. injection of *Escherichia coli* LPS (0111:B4, Sigma, L4391) at 25 mg/kg body weight and were monitored for survival. For short-term studies, mice were sacrificed 18 h post LPS i.p. injection, followed by blood collection with cardiac puncture. Peritoneal cavity was then washed with PBS and spleens were collected.

### Macrophage Preparation

For BMDM preparation, bone marrow cells were flushed from the femurs and tibiae of mice and cultured in complete RPMI in the presence of 20% medium conditioned by L929 mouse fibroblasts, as described (21). On day 7, BMDMs were split and used as indicated. To isolate thioglycollate-elicited macrophages, mice were i.p. injected with 1.5 ml of 5% thioglycollate and peritoneal macrophages were flushed out 3 days post-injection.

### Cytokine Detection

Isolated peritoneal macrophages (85% pure CD11b^+^/F4.80^+^ macrophages) were seeded on six-well plates at 3 × 10^6^ cells/well. Unstimulated and LPS-stimulated cells were cultured for indicated period. Supernatants were subsequently collected and stored at −20°C until analysis. TNFα and IL-6 ELISA kits were purchased from R&D Systems and performed as per the manufacturers' instructions and read at 450 nm by a SpectraMax microplate reader (Molecular Devices, Sunnyvale, CA).

### Flow Cytometry

To examine macrophages by flow cytometry, cells were analyzed from either the spleen or peritoneal cavity and gated on CD11b^+^ and F4/80^+^ double positive cells (Supplemental Figure 2). Briefly, spleens were isolated and disrupted to a single cell suspension between two frosted slides in RPMI supplemented with 10% FCS, 4 mM L-glutamine, 10 U/ml penicillin, 100 μg/ml streptomycin, 0.5 mM 2-ME. Cells were then washed and resuspended in FACS buffer (PBS supplemented with 2% FBS) and stained with indicated flow cytometry antibodies. For analysis of peritoneal macrophages and neutrophils, euthanized animals were injected with 5 ml ice cold PBS supplemented with 3% FBS into the peritoneum following the removal of the outer layer of skin exposing the peritoneal lining. The abdomen was next massaged and the fluid was withdrawn using a 25 G needle, bevel up. The cells were washed, resuspended in FACS buffer, and stained with indicated flow cytometry antibodies.

To analyze macrophage surface phenotype, macrophages were incubated in the presence or absence of LPS. Cells were then harvested, stained, and gated on CD11b-conjugated FITC (Clone: M1/70) and F4.80-conjugated APC with the following per triplicate group: Annexin V (BD Biosciences), CD80 (Clone: 16-10A1), CD86 (Clone: GL-1), CD40 (Clone: 3/23), MHCII-I-Ab (Clone: AF6-120.1), PD-L1 (Clone: MIH5), PD-L2 (Clone: TY25). The total numbers of cells expressing the indicated phenotypes were determined by factoring the total cell counts (Supplemental Figure 1) with the percent expression of gated markers, taking into account the parent population gates.

Analysis of neutrophils from the spleen and peritoneum (cell preparation described above) were gated on Gr-1 conjugated FITC (RB6-8C5) and CD11b-conjugated PerCP/Cy5.5 (M1/70) (Supplemental Figure 2).

Cytokine measurement by flow cytometry was analyzed by gating on macrophages (CD11b^+^ and F4/80^+^ double positive cells) or neutrophils (Gr-1^+^ and CD11b^+^ double positive cells) following the incubation of splenocytes or peritoneal cells with golgi block for 6 h. Cells were stained with TNFα-conjugated APC (MP6-XT22) and IL-6 (MP5-20F3). Total number of cells producing indicated cytokine was calculated by factoring the total number of cells indicated in **Table 3** by the percent expression of gated markers, taking into account the parent population gates.

Propidium iodide was obtained from BD bioscience and used according to the manufacturers' instructions. All flow cytometry Abs were purchased from eBiosciences. Stained cells were then analyzed with an Accuri C6 Flow Cytometer (BD Biosciences).

### ATPlite Assay

BMDM cells were seeded into 96-well plates with 5,000 cells per well in triplicate and treated with various concentrations of LPS for 3 days followed by ATPlite assay using an ATPlite kit (Perkin Elmer) according to the manufacturer's instructions.

### Western Blot Analysis

BMDM cells stimulated with LPS for different time periods were harvested, lysed and subjected to Western blotting, using various antibodies as follows: Sag monoclonal mouse antibody was raised against the RING domain (AA44-113) (22), Roc1 polyclonal rabbit antibody (23), pho-IκBα (Cell Signaling), IκBα (Santa Cruz), p65 (Santa Cruz), Parp (Cell Signaling), Procaspase-3 (Santa Cruz), and actin (Sigma), as a loading control.

### Nuclear-Cytoplasmic Fractionation

Cells were treated with or without LPS (100 ng/ml) as control for 1 h, then cells were washed with cold PBS and collected by scraping followed by the addition of lysis buffer A [10 mM HEPES, pH 8.0, 10 mM NaCl, 1.5 mM MgCl_2_, 1 mM DTT, 0.1% NP-40 and a protease inhibitor cocktail (Roche)]. Lysis was completed on ice for 10 min. Supernatants containing the cytoplasmic fractions were collected after centrifugation (400 g, 4°C, 5 min). The pellets were washed three times in lysis buffer A and then lysed in buffer B (20 mM HEPES, pH 8.0, 20% glycerol, 500 mM NaCl, 1.5 mM MgCl_2_, 0.2 mM EDTA, pH 8.0, 1 mM DTT, and a protease inhibitor cocktail) for 30 min on ice, then centrifuged at 15,000 g, 4°C for 15 min. The supernatants containing nuclear lysates were collected.

### Neutrophil Isolation From Blood

Blood was carefully layered over neutrophil separation media, followed by centrifugation at 500 g for 35 min at room temperature. The layer of neutrophils and all of the isolation media beneath the neutrophils were carefully collected and then diluted to 10 ml with HBSS without Ca^2+^/Mg^2+^. Cells were suspended and then collected by centrifugation at 350 g for 10 min. The residual red blood cells were lysed by Red Cell Lysis Buffer (Roche), followed by centrifugation at 250 g for 5 min.

### Microarray Analysis

Mice were i.p. injected with PBS or LPS (25 mg/kg body weight). The bone marrow cells were flushed out 6 h post injection, followed by RNA extraction and subsequent microarray analysis using Mouse Gene ST 2.1 Strip (Affymetrix). The expression values for each gene was calculated using a robust multi-array average (24). Probeset that had a 2-fold or greater change with the added constraint that the expression value of one of the two samples was 2^3^ or greater were selected. The expression of genes was compared in WT or KO bone marrow in response to LPS stimulation. Gene list that only changed in KO were enhanced using GO enrichment analysis of ConsensusPathDB (25). The expression heatmap of selected gene list were generated under R (version 3.4.0).

### Gene Expression

Total RNA was isolated from cells with Trizol reagent (Invitrogen). Complementary DNA was made from RNA with Superscript III (Invitrogen) and subjected to reverse transcription PCR or quantitative real-time PCR (qRT-PCR) with a 7,500 Real Time PCR system (Applied Biosystems). The cycling program for qRT-PCR was set as follows: 50°C 2 min, 95°C 10 min for the PCR initial activation and 45 cycles of denaturation at 95°C for 15 s, annealing and extension at 60°C for 1 min. The sequences of Sag, TNFα, IL-6, Mpo, Elane, LitaF, and GAPDH are as follows: Sag-Fwd: 5′- CGC TGA GCC ACC GTA CCT - 3′, Sag-Rev: 5′- TTA CAC TCT CCC CAG ACC ACA A - 3′; TNFα-Fwd: 5′- CCC CAA AGG GAT GAG AAG TT - 3′, TNFα-Rev: 5′- CTT GGT GGT TTG CTA CGA CG - 3′; IL6-Fwd: 5′- TCA TAT CTT CAA CCA AGA GGT AAA A - 3′, IL6-Rev: 5′- CGC ACT AGG TTT GCC GAG TA - 3′; Mpo-Fwd: 5′- ATC ACG GCC TCC CAG GAT AC - 3′, Mpo-Rev: 5′- GTT GTT GGG CGT GCC ATA TT - 3′, Elane-Fwd: 5′- CAG GCA TCT GCT TCG GGG A - 3′, Elane-Rev: 5′- GGG ATG GGT AAG AAG GTG GTC A - 3′, LitaF-Fwd: 5′- CAG TCT GTG TCT GCT GGG ATG C - 3′, LitaF-Rev: 5′- ACT ACC TCT GCA GTG GCG GG - 3′, GAPDH-Fwd: 5′- GCC GCC TGG AGA AAC CTG CC - 3′, GAPDH-Rev: 5′- GGT GGA AGA GTG GGA GTT GC - 3′.

### Statistical Analysis

Bars and error bars represent the means and standard errors of the mean, respectively. The two-tailed Student *t-* test was used for the comparison of parameters between groups. Survival analysis was performed by Kaplan-Meier analysis. Statistical significance was determined as ^*^p < 0.05; ^**^p < 0.01; ^***^p < 0.001.

## Results

### Characterization of LysM-Cre/*Sag^*fl*/*fl*^* Mice

**Figure 1:**
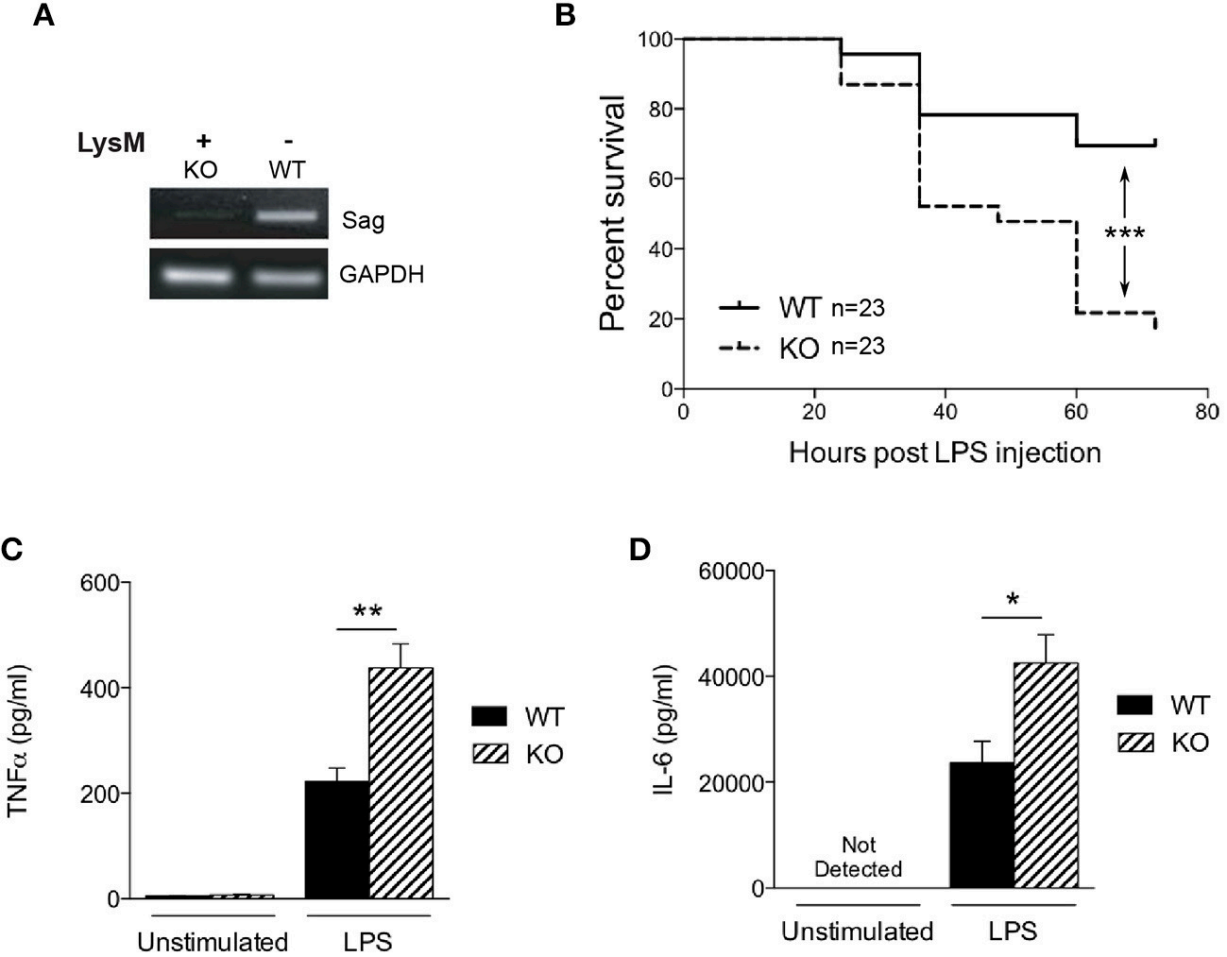
Characterization of LysM-Cre/*Sag*^*fl*/*fl*^ mice. **(A)** Sag expression is significantly decreased in peritoneal macrophages from LysM-Cre^+^/*Sag*^*fl*/*fl*^ mice. Residential peritoneal macrophages were collected from LysM-Cre^−^/*Sag*^*fl*/*fl*^ wild type (WT) or LysM-Cre^+^/*Sag*^*fl*/*fl*^ knockout (KO) mice and mRNA was extracted for RT-PCR. **(B)** WT and KO mice were injected i.p. with LPS (25 mg/kg body weight), and their survival was monitored and plotted every 12 h up to 72 h. *n* = 23 for WT or KO group. ^***^p < 0.001, log-rank test. **(C)** TNFα and **(D)** IL-6 in the cardiac sera of mice 18 h after LPS injection; measured by ELISA. ^*^p < 0.05; ^**^p < 0.01, ^***^p < 0.001.

To determine the role of SAG in the innate inflammatory response—specifically macrophages—we crossed *Sag*^*fl*/*fl*^ mice with LysM-Cre transgenic mice, which express Cre recombinase in myeloid cells (26). Inactivation of *Sag* was verified in macrophages of the peritoneal cavity by RT-PCR analysis (Figure 1A). The mRNA levels of *Sag* were significantly decreased in resident peritoneal macrophages from LysM-Cre^+^/*Sag*^*fl*/*fl*^ (KO) mice compared to LysM-Cre^−^/*Sag*^*fl*/*fl*^ (WT) mice. To determine whether hematopoietic deficiency of Sag in LysM^+^ cells affects their development and/or other cells, we performed serial complete blood count (CBC) analysis of mice at 4 and 12 weeks of age. Sag-deficiency in the myeloid lineage had no significant effect on the total cell numbers of white blood cells, neutrophils, lymphocytes, monocytes, eosinophils, basophils, red blood cells, and platelets in the peripheral blood (Table 1).

**Table 1:** Mouse CBC.

**Parameter**	**Unit**	**4 Weeks**			**12 Weeks**		
		**WT (LysM^−^)**	**KO (LysM^+^)**	***P* value**	**WT (LysM^−^)**	**KO (LysM^+^)**	***P* value**
WBC#	K/μL	3.72 ± 0.07	4.08 ± 1.52	0.65	7.67 ± 1.24	7.64 ± 3.27	0.88
NE#	K/μL	0.54 ± 0.13	0.42 ± 0.07	0.11	1.98 ± 1.08	1.96 ± 1.00	0.52
LY#	K/μL	2.99 ± 0.17	3.38 ± 1.34	0.59	5.01 ± 0.89	5.08 ± 1.83	0.67
MO#	K/μL	0.16 ± 0.03	0.26 ± 0.14	0.24	0.38 ± 0.09	0.49 ± 0.33	0.47
EO#	K/μL	0.02 ± 0.03	0.02 ± 0.02	0.99	0.23 ± 0.37	0.08 ± 0.11	0.15
BA#	K/μL	0.00 ± 0.01	0.00 ± 0.00	0.88	0.07 ± 0.05	0.04 ± 0.06	0.54
RBC	M/μL	5.70 ± 0.26	6.66 ± 2.12	0.40	9.41 ± 2.10	10.55 ± 0.88	0.41
HB	g/dL	8.38 ± 0.58	9.46 ± 2.73	0.47	12.97 ± 2.64	14.7 ± 0.42	0.31
HCT	%	29.68 ± 1.63	33.86 ± 10.03	0.44	48.43 ± 11.03	52.75 ± 2.52	0.64
PLT	K/μL	469.8 ± 51.9	543.4 ± 150.4	0.39	689.7 ± 126.3	725.5 ± 165.4	0.82
MPV	fL	3.82 ± 0.26	4.18 ± 0.60	0.31	4.23 ± 1.22	4.12 ± 0.33	0.31

*WBC, absolute number of white blood cells; NE, absolute number of neutrophils; LY, absolute number of lymphocytes; MO, absolute number of monocytes; EO, absolute number of eosinophils; BA, absolute number of basophils; RBC, absolute number of red blood cells; HB, hemoglobin; HCT, hematocrit; PLT, absolute number of platelets; MPV, mean platelet volume. Shown are mean ± SD. n = 4 for WT, n = 5 for KO*.

Next, we determined whether Sag deficiency affected the phenotype of macrophages by performing immunophenotypical analysis of macrophages isolated from the spleen, bone marrow, and peripheral lymph nodes. No significant differences were observed between WT and KO animals in either the absolute numbers or percentages of macrophages (CD11b^+^ and F4/80^+^) or the expression of co-stimulatory molecules (MHCII, CD80, CD86, CD40, PD-L1, PD-L2 (Table 2 and Supplemental Figure 1). These data indicate that targeted deletion of *Sag* in the myeloid lineage does not result in significant altered development, total numbers, or phenotype of these cells.

**Table 2:** Mouse naïve phenotype.

	**Marker**	**WT (LyzM^−^)**	**KO (LyzM^+^)**	***P* value**
Spleen	Total count	4.18e7 ± 7.10e6	4.47e6 ± 1.25e6	0.60
	CD11b/F4.80	1.81e6 ± 1.96e5	1.95e6 ± 1.37e5	0.55
	MHCII	1.53e6 ± 1.53e5	1.65e6 ± 1.11e5	0.66
	CD80	1.29e6 ± 2.01e5	1.41e6 ± 1.29e5	0.50
	CD86	1.07e6 ± 1.70e5	1.21e6 ± 8.09e4	0.88
	CD40	1.89e5 ± 6.99e4	1.77e5 ± 2.67e4	0.67
	PDL1	1.22e6 ± 1.56e5	1.31e6 ± 1.09e5	0.90
	PDL2	3.08e5 ± 9.94e4	3.23e5 ± 1.10e4	0.60
Bone marrow	Total count	2.64e7 ± 2.01e6	2.32e7 ± 2.95e6	0.89
	CD11b/F4.80	7.60e6 ± 1.19e6	7.80e6 ± 4.70e5	0.48
	MHCII	2.28e6 ± 4.01e5	1.93e6 ± 2.19e5	0.87
	CD80	1.21e6 ± 2.43e5	1.25e6 ± 1.28e5	0.42
	CD86	1.14e6 ± 2.84e5	1.40e6 ± 3.53e4	0.20
	CD40	1.80e5 ± 1.99e4	2.56e5 ± 4.59e4	0.62
	PDL1	6.41e5 ± 1.34e5	7.31e5 ± 9.92e4	0.11
	PDL2	1.73e5 ± 3.61e3	3.21e5 ± 7.12e3	0.89
Peripheral lymph nodes	Total count	6.29e6 ± 1.69e6	8.39e6 ± 5.87e5	0.81
	CD11b/F4.80	2.67e5 ± 5.14e4	2.81e5 ± 1.82e4	0.79
	MHCII	2.60e5 ± 5.04e4	2.75e5 ± 1.93e4	0.59
	CD80	1.25e5 ± 2.58e4	1.42e5 ± 1.21e4	0.79
	CD86	2.07e5 ± 2.86e4	2.20e5 ± 2.28e4	0.51
	CD40	3.43e4 ± 9.63e3	2.64e4 ± 5.26e3	0.67
	PDL1	2.01e5 ± 3.72e4	2.19e5 ± 1.29e4	0.60
	PDL2	7.06e4 ± 1.89e4	5.81e4 ± 1.10e4	0.81

*Shown are mean ± SD. n = 8 for WT, n = 8 for KO*.

### The *in vivo* Responses of Sag-Deficiency in Myeloid Lineage to LPS

Previous studies have shown that SAG knockdown in macrophages results in decreased release of inflammatory cytokines upon LPS stimulation (9, 10). Therefore, we next tested the hypothesis whether *in vivo* LPS stimulation would result in decreased proinflammatory cytokines and decreased mortality in KO animals. We injected intraperitoneally (i.p) WT and KO littermate mice with a lethal dose of LPS (25 mg/kg). To our surprise, the survival study demonstrated significantly increased mortality in KO mice with Sag deficient myeloid cells (Figure 1B). We next measured LPS-induced proinflammatory cytokines production and observed that the levels of TNFα and IL-6 in the sera of KO mice were significantly higher than those of WT mice at 18 h after LPS injection (Figures 1C,D). These surprising data suggested that mice with LysM Sag deficiency were hypersensitive to LPS.

### The *in vitro* Responses of Sag-Deficient Macrophages to LPS

**Figure 2:**
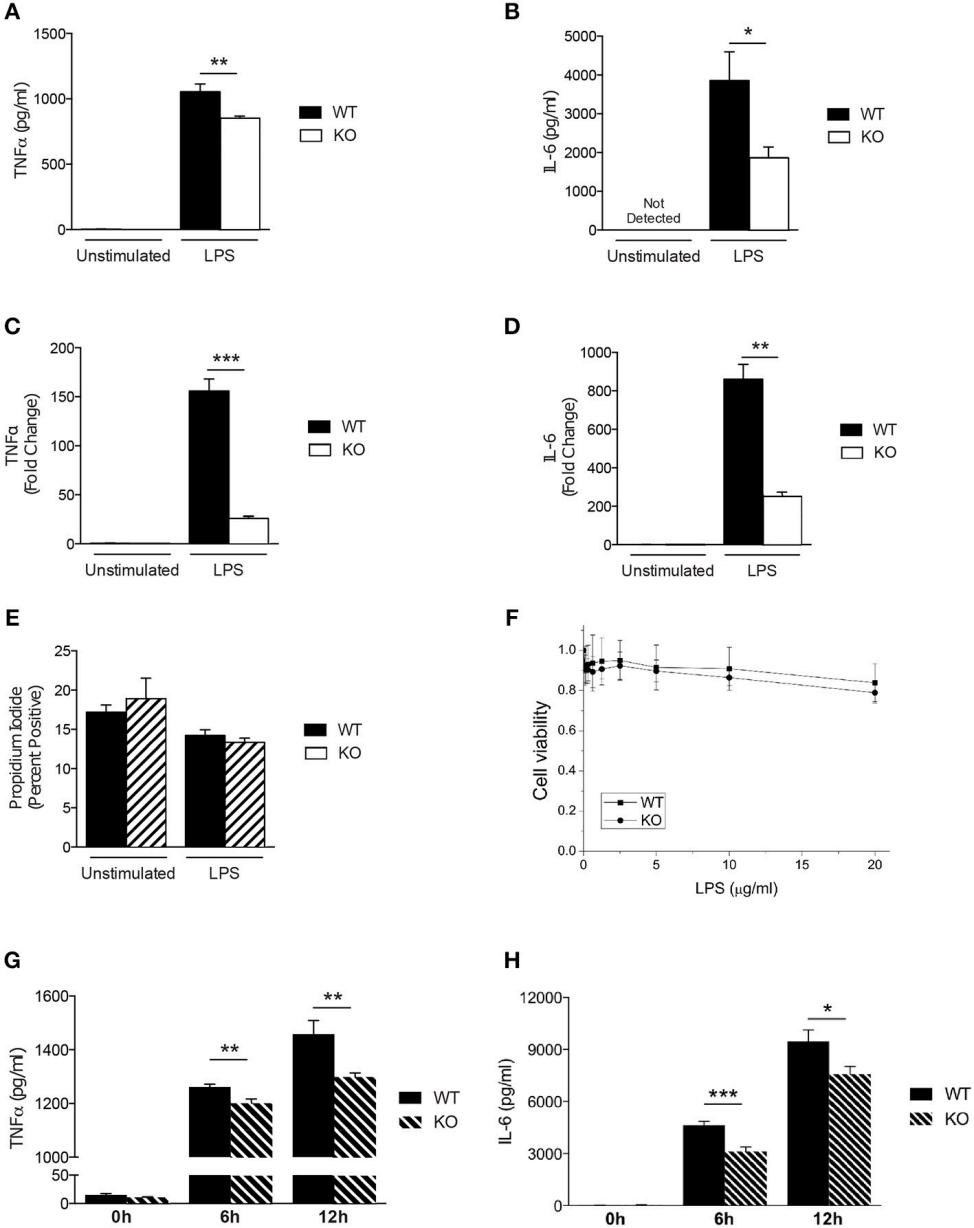
Characterization of Sag-deficient macrophages *in vitro*. **(A–D)** Reduced production of proinflammatory cytokines in thioglycollate-elicited peritoneal macrophages from LysM-cre/*Sag*^*fl*/*fl*^ mice. Cells culture supernatants were analyzed from either unstimulated or stimulated macrophages with 100 ng/ml LPS for 24 h. TNFα and IL-6 protein concentrations were measured by **(A,B)** ELISA and **(C,D)** mRNA transcripts in cells by qPCR. **(E,F)** Sag deficiency has no effect on the **(E)** apoptosis (Annexin V/PI) of peritoneal macrophages or **(F)** viability of BMDMs in response to LPS. The peritoneal macrophages derived from LysM-cre/*Sag*^*fl*/*fl*^ mice were treated with 100 ng/ml LPS for 72 h **(E)**. The viability of BMDMs treated with various concentrations of LPS for 72 h in triplicates were normalized to untreated cells and shown as mean ± SEM (*n* = 2) **(F)**. **(G,H)** Reduced production of proinflammatory cytokines in BMDMs derived from LysM-cre/*Sag*^*fl*/*fl*^ mice. BMDMs were stimulated with 100 ng/ml LPS for 6 and 12 h. **(G)** TNFα and **(H)** IL-6 release were measured in cell culture supernatants by ELISA. ^*^p < 0.05; ^**^p < 0.01; ^***^p < 0.001.

Previous studies have demonstrated that SAG regulates other innate immune cell responses, such as DCs (16) and macrophages during infection (9). We next analyzed the response of Sag-deficient peritoneal macrophages stimulated with LPS. We observed that the release of the proinflammatory cytokines TNFα and IL-6 were significantly reduced by Sag-deficient macrophages (Figures 2A,B) with a similar decrease in mRNA transcripts for TNFα and IL-6 (Figures 2C,D) from these cells.

Studies have shown that inactivation of SAG reduces the viability of tumor cells and infected murine macrophages *in vitro* (9, 27). However, primary bone marrow derived DCs did not exhibit increased apoptosis upon inhibition of SAG E3 ligase complex (16) suggesting that inactivation of SAG in healthy, non-cancerous cells may not affect cell survival. Thus, we next tested whether primary WT and Sag-deficient macrophages stimulated with LPS resulted in altered cell viability. Once again, we observed no difference between WT and Sag-deficient peritoneal macrophages (Figure 2E).

To ensure that the results observed were not specific to macrophages isolated from a particular compartment, we next examined bone marrow derived macrophages (BMDMs). Consistent with our results from peritoneal macrophages, no significant differences in viability of BMDMs from WT or KO mice were observed (Figure 2F). Furthermore, LPS-induced release of TNFα and IL-6 cytokines by BMDMs over a total time period of 12 h, was significantly reduced in the absence of Sag (Figures 2G,H). These data together suggest that Sag deficiency in macrophages significantly impairs the production of proinflammatory cytokines in response to LPS stimulation, but had no significant effect on the viability.

### Sag Deficient Macrophages Release Less Cytokines *in vivo*

**Figure 3:**
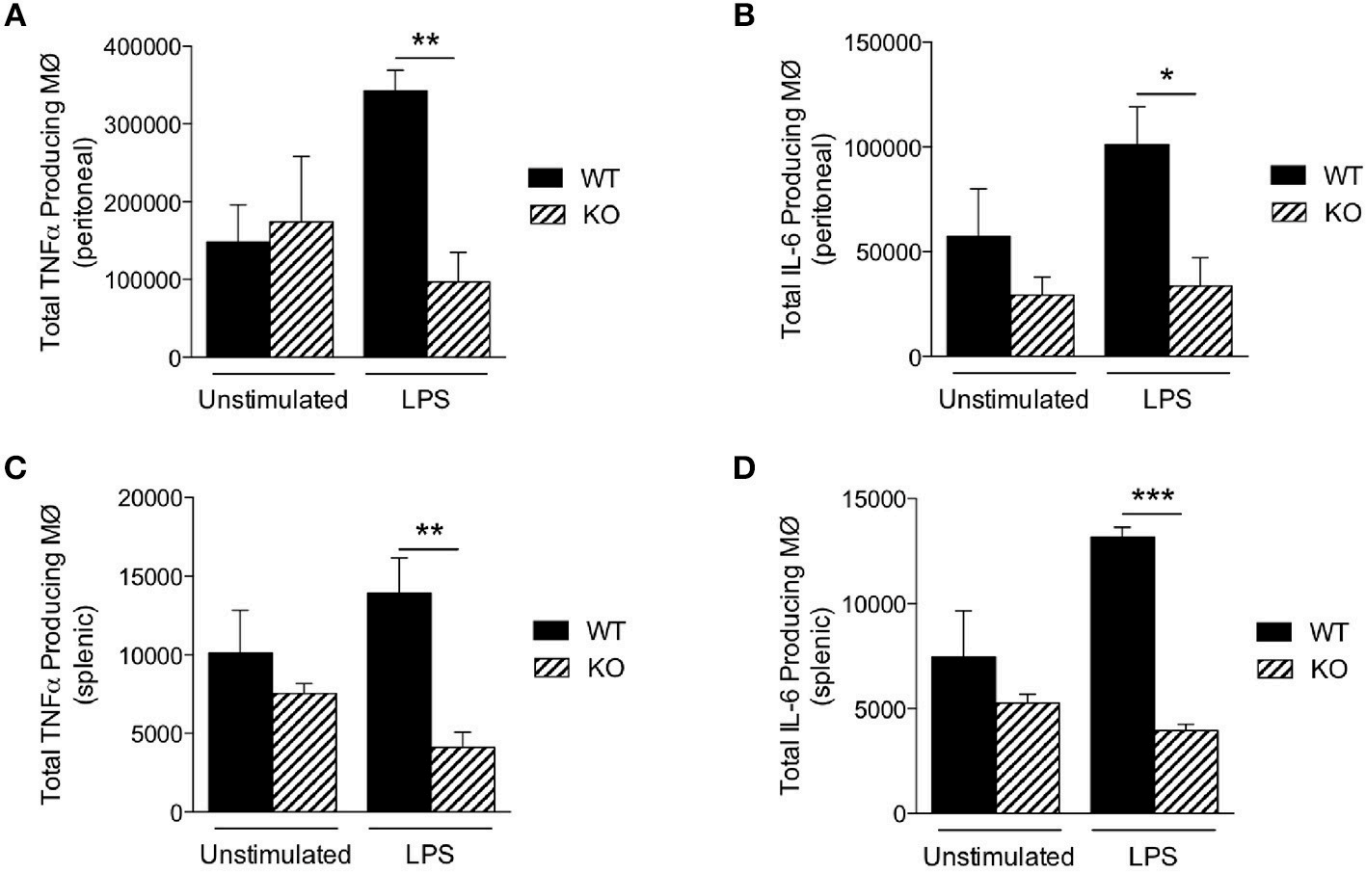
Sag-deficient macrophages release less cytokines *in vivo*. LysM-cre/*Sag*^*fl*/*fl*^ (KO) and *Sag*^*fl*/*fl*^ (WT) mice were i.p. injected with PBS or LPS (25 mg/kg body weight). The cells were harvested 18 h post injection. The total numbers of macrophages producing **(A,C)** TNFα and **(B,D)** IL-6 in the **(A,B)** peritoneal cavity and **(C,D)** spleen were determined by flow cytometry. *N* = 4 for PBS elicited; *N* = 6 for LPS elicited. ^*^p < 0.05; ^**^p < 0.01; ^***^p < 0.001.

To further explore why KO mice were hypersensitive to LPS, we next measured LPS-induced production of proinflammatory cytokines *in vivo*. We injected LPS intraperitoneally (25 mg/kg) and then analyzed macrophages of the peritoneum and spleen by flow cytometry 18 h later. However, although the total numbers of macrophages were comparable between WT and KO mice (Table 3), the numbers of TNFα and IL-6 producing macrophages from the peritoneum (Figures 3A,B) and spleen (Figures 3C,D) were significantly lower in KO animals, compared to WT (Figure 3). Taken together, these data suggest that in the absence of Sag in myeloid cells, mice show (1) increased LPS-induced mortality, (2) increased proinflammatory cytokines in the sera, but (3) the increased mortality was not due to increased cytokine release from the macrophages. Thus, these data indicated that the increase in proinflammatory cytokines contributing to mortality might be from another type of myeloid cell capable of responding to LPS and secreting increased proinflammatory cytokines.

**Table 3:** Elicited macrophages.

**Organ**	**Marker**	**WT (LyzM^−^)**	**KO (LyzM^+^)**	***P* value**
Peritoneal macrophages	PBS elicited	3.69e6 ± 4.88e5	4.71e6 ± 9.13e5	0.43
	LPS elicited	2.19e6 ± 9.11e5	2.19e6 ± 2.55e5	0.99
Splenic macrophages	PBS elicited	8.04e7 ± 1.80e7	7.14e7 ± 2.60e6	0.66
	LPS elicited	9.30e7 ± 1.24e7	8.73e7 ± 1.31e7	0.82

*Shown are mean ± SD. n = 4 for PBS elicited, n = 6 for LPS elicited*.

### Impact of Sag Deficiency in Macrophages: IκB Accumulation

**Figure 4:**
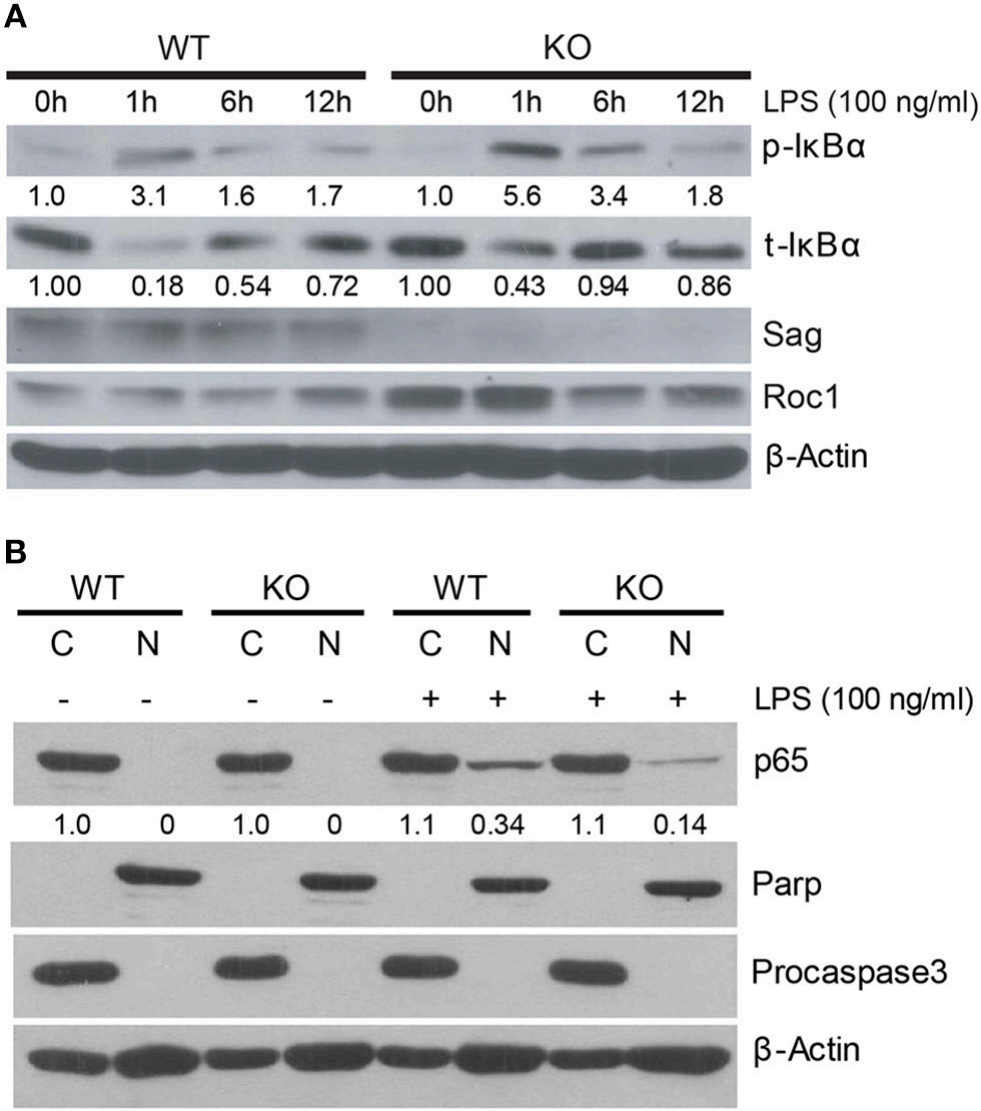
IκB accumulation in Sag-deficient macrophages. **(A)** Bone marrow derived macrophages (BMDMs) from WT (*n* = 4) and KO (*n* = 4) mice were pooled by group and stimulated with 100 ng/ml LPS for indicated time periods, followed by immunoblot with indicated Abs. Densitometric analysis is shown underneath the blots after normalization with β-actin. **(B)** Western blot analysis of p65 isoform of NF-κB protein using nuclear [N] and cytosolic [C] fractions, extracted from peritoneal WT and KO macrophages that were stimulated concurrently with LPS (100 ng/ml) for 1 h. Densitometric analysis of p65 level in the nuclear fraction vs. cytosolic fraction is shown underneath the blot. Parp and Procaspase-3 serve as biomarkers of nuclear and cytoplasmic fractions, respectively.

We next pursued the mechanism by which *Sag* deletion causes reduction of proinflammatory cytokines with focus on IκBα/NF-κB, since our previous studies have shown that IκBα is a direct substrate of SAG E3 ligase (11). SAG disruption prevents the activation and translocation of NF-κB, which contributes to decreased inflammatory cytokines from DCs and to the increased radiosensitivity observed in Sag-null murine embryonic stem cells (11, 16). Upon stimulation of a cell by TLR4, IκBα is phosphorylated and degraded as a direct substrate of SAG- SCF^β−*TrCP*^ E3 ubiquitin ligase (11). To determine if IκBα and NF-κB play a similar role in macrophages as was previously shown in dendritic cells (16), we examined the protein levels of phosphorylated and total IκB in macrophages and found that LPS stimulation resulted in accumulation of phosphorylated IκBα as well as total IκBα in Sag-deficient macrophages (Figure 4A), indicating that the degradation of IκB was impaired in the absence of Sag. Consistent with this notion, we found that the translocation of p65 NF-κB from the cytoplasm to the nucleus was reduced in Sag-deficient macrophages compared to Sag-competent WT controls, as shown in Figure 4B, in which Parp and Procaspase-3 serve as biomarkers of nuclear and cytoplasmic fractions, respectively. These data suggest that similar to the effects seen in DCs, Sag disruption affects LPS-induced IκBα degradation, which inhibits NF-κB activation in macrophages, leading to reduced levels of proinflammatory cytokines, such as TNFα and IL-6.

### Sag Deficiency Increases Proinflammatory Responses by Neutrophils

**Figure 5:**
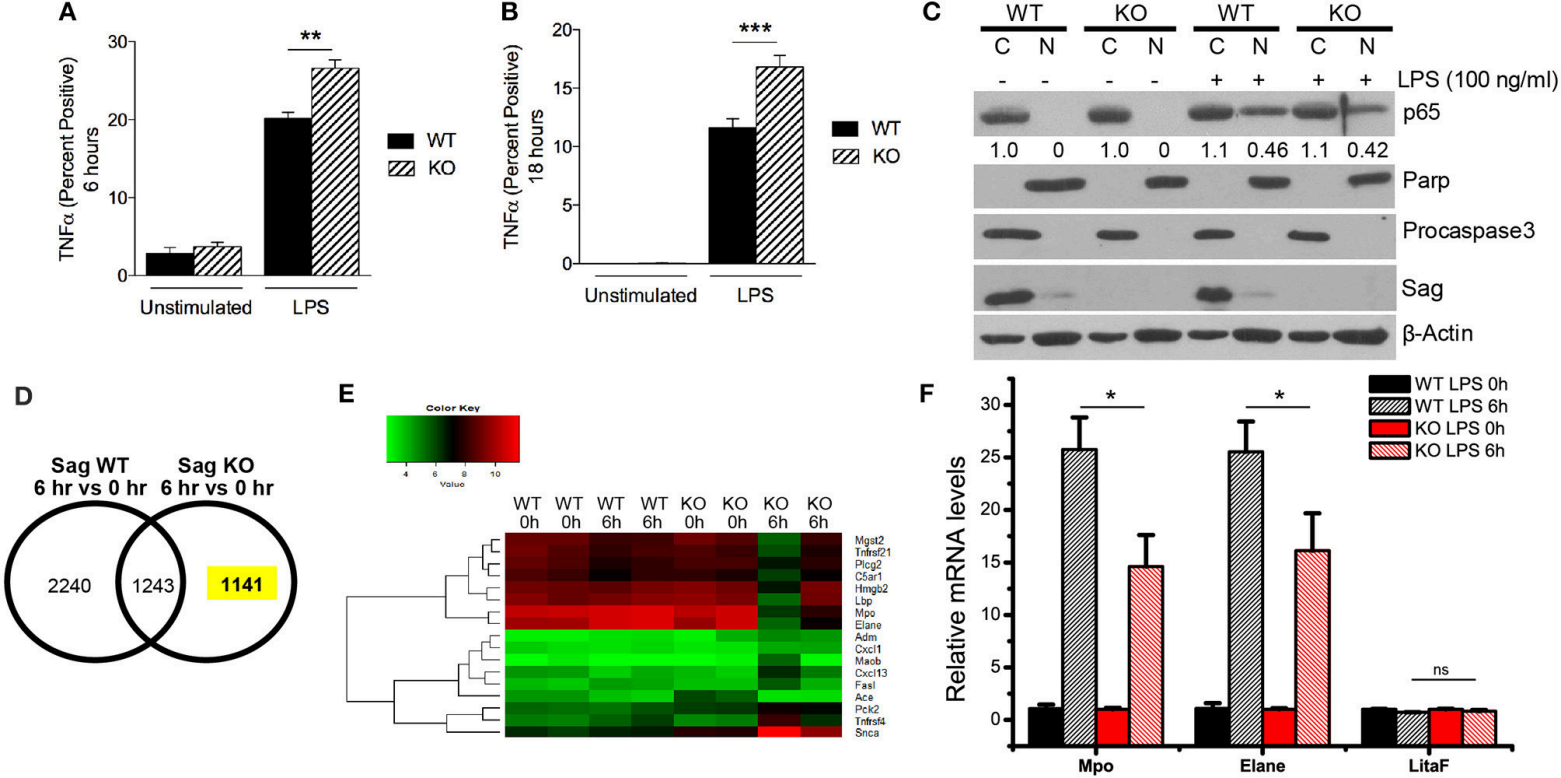
Increased TNFα and decreased induction of genes responsive to LPS in Sag-deficient neutrophils and bone marrow. **(A,B)** Splenocytes from LysM-Cre^−^/*Sag*^*fl*/*fl*^ (WT) and LysM-Cre^+^/*Sag*^*fl*/*fl*^ (KO) mice (*n* = 5 for each genotype) were stimulated with LPS (100 ng/ml) and TNFα production by CD11b^+^ Gr-1^+^ neutrophils was analyzed by flow cytometry **(A)** 6 h and **(B)** 18 h following stimulation. **(C)** Protein analysis by western blot of p65 isoform of NF-κB protein using nuclear [N] and cytosolic [C] fractions of neutrophils isolated from blood of WT and KO mice stimulated concurrently with LPS (100 ng/ml) for 1 h. Densitometric analysis of p65 NF-κB presence in the nuclear fraction and cytosolic fraction is shown underneath the blot. Parp and Procaspase-3 serve as biomarkers of nuclear and cytoplasmic fractions, respectively. **(D,E)** Affymetrix microarray analysis upon stimulation with LPS for 6 h: Bone marrow cells were isolated from two individual WT and KO mice with i.p. injection of PBS or LPS (25 mg/kg body weight) for 6 h, followed by RNA extraction and Affymetrix microarray analysis. **(D)** The expression of genes was compared in WT or KO bone marrow in response to LPS stimulation. **(E)** GO enrichment analysis was performed in a total of 1,141 genes whose expression was specifically altered in KO bone marrow in response to LPS stimulation. The expression heatmap of genes annotated with term “response to lipopolysaccharide” was generated. **(F)** Validation of microarray results by qRT-PCR: Bone marrow was isolated from three individual WT and KO mice i.p. injected with PBS or LPS (25 mg/kg body weight) for 6 h, followed by RNA extraction and qRT-PCR analysis. ^*^p < 0.05; ^**^p < 0.01; ^***^p < 0.001; ns, not significant.

Similar to macrophages, neutrophils are myeloid lineage cells, which also express LysM. Thus, to determine the origin of increased LPS-induced cytokine production in the sera of mice with Sag deficient myeloid lineage cells, we next analyzed the response of neutrophils (CD11b^+^ and Gr-1^+^) to LPS stimulation. We stimulated whole splenocyte cultures with LPS for 6 h (Figure 5A) and 18 h (Figure 5B) and observed a significant increase of TNFα expression in Sag-deficient CD11b^+^ Gr-1^+^-neutrophils (Figures 5A,B).

As NF-κB is the transcription factor for TNFα, we next examined the translocation of NF-κB from the cytoplasm to the nucleus in neutrophils. In contrast to the results seen in macrophages (Figure 4B), we did not find a difference in the translocation of NF-κB in neutrophils (Figure 5C). These data suggested that Sag had a differential role in neutrophils, as compared with macrophages.

Previous studies have shown that LPS stimulation activates several intracellular signaling pathways including NF-κB pathways in macrophages (28). To explore why Sag deficiency in neutrophils causes an increase in TNFα release in contrast to what was observed in macrophages, we analyzed the mRNA transcripts in bone marrow cells from LysM-Cre^−^/*Sag*^*fl*/*fl*^ (WT) and LysM-Cre^+^/*Sag*^*fl*/*fl*^ (KO) mice in response to LPS. Sag deficiency was first confirmed in the bone marrow from KO mice by RT-PCR analysis (Supplemental Figure 3A). Microarray analysis revealed that the expression of 1,141 genes was specifically altered in response to LPS in Sag-deficient bone marrow cells (Figure 5D). Gene ontology (GO) analysis was then performed to enrich several sets of genes associated with multiple processes and pathways (Supplemental Figure 3B), including a list of genes responsive to LPS (Figure 5E, Supplemental Figure 3C). More importantly, we validated the microarray results by qRT-PCR and found that the induction of Mpo, the major enzyme to mediate the protective function of neutrophils from LPS-induced toxicity (17), and Elane, Neutrophil expressed elastase, was remarkably decreased in bone marrow cells from KO mice after LPS stimulation (Figure 5F). Furthermore, qRT-PCR results also confirmed that the expression of LitaF (LPS-induced TNFα factor), an important mediator of LPS-induced inflammatory response (29), but not shown in the gene list (Supplemental Figure 3C), had no difference between bone marrow from WT and KO mice (Figure 5F).

We next determined whether other cells that did not express LysM enzyme were unaffected. We, therefore, stimulated WT and KO bone marrow derived DCs (BMDCs) with LPS and examined the production of TNFα and IL-6, and found no difference in either the release of TNFα (Supplemental Figure 4A) and IL-6 (Supplemental Figure 4B) or in the mRNA expression of these proinflammatory cytokines (Supplemental Figures 4C,D). These data indeed suggested that Sag deficiency in LysM^+^ cells did not affect non-LysM expressing cells.

## Discussion

Our data have shown that although Sag deficiency in LysM^+^ mice has no effect on the total CBC including myeloid cells of the innate immune system, specifically macrophages and polymorphonuclear leukocytes (PMNs) (Table 1), a significant LPS-induced increase in proinflammatory cytokines in the sera, and more importantly, an increase in LPS-induced mortality were observed in KO mice. To elucidate underlying mechanism, we explored cell (macrophages vs. neutrophils) -specific responses to LPS. Upon LPS stimulation, Sag deficient macrophages released less proinflammatory cytokines due to reduced IκB degradation and a subsequent decrease in NF-κB translocation to the nucleus. These results were thus incapable of explaining the enhanced LPS induced mortality observed in KO mice. In contrast, LPS stimulated Sag-deficient neutrophils released more TNFα with a reduced induction of Mpo and Elane, two cytoprotective proteins against bacterial endotoxin that are specifically expressed in neutrophils (17, 18), thus contributing to increased LPS mortality.

More specifically, previous studies (both ours and others) have shown that SAG and SAG-dependent neddylation play a critical role in regulation of the inflammatory response involving several immune cells, including macrophages (9), dendritic cells (16), and T-cells (30), and interestingly, in human hepatocellular carcinoma cells as well (31, 32). Specifically, inactivation of Sag by siRNA-mediated knockdown or chemical inhibition of neddylation with MLN4924, a potent small molecular inhibitor of NEDD8 activating enzyme E1 NAE that effectively blocks cullin neddylation to inhibit CRL activation (33), impaired inflammatory response. In addition, hemizygous knockout of Cullin-5, the substrate of SAG-mediated neddylation and a scaffold component of SAG-CRL5 E3 ubiquitin ligase complex, also reduced proinflammatory cytokine production in sera and improved mice survival in response to LPS stimulation by repressing TRAF6 polyubiquitination to impair TRAF6-dependent NF-κB activation (34). Consistently, our *in vivo* study demonstrated that the deficiency of Sag suppressed the production of proinflammatory cytokines in macrophages (Figures 2, 3). By contrast, we observed significantly increased TNFα production in Sag-deficient CD11b^+^ Gr-1^+^-neutrophils (Figures 5A,B) and a net increase of proinflammatory cytokine production in the sera from LysM-Cre^+^/*Sag*^*fl*/*fl*^ mice after LPS stimulation (Figures 1C,D). The reasons for the differential response to Sag deficiency in macrophages and neutrophils are unclear at the present time. We propose that these responses are likely due to differences in cell context in which different Sag substrates are targeted for degradation in a cell-type specific manner. Indeed, the degradation of IκBα is inhibited in macrophages (Figure 4A) and dendritic cells (16), leading to the inhibition of NF-κB nuclear translocation, which is not seen in neutrophils upon *Sag* deletion (Figure 5C). It is worth noting that MLN4924 was recently shown to inhibit the production of proinflammatory cytokines in response to LPS in purified peritoneal neutrophils (35). However, MLN4924 pretreatment followed by LPS stimulation for 4 h had no significant effects on the viability of neutrophils in that study.

Innate immune signaling is poorly understood. Our finding that Sag protein plays a differential role in macrophages and neutrophils is significant and novel as it is the first demonstration that Sag function can be determined by targeting—or not targeting—IκBα in two types of critical cells of the innate immune system. This observation is consistent with our recent finding that Sag plays a tissue specific role during Kras-triggered tumorigenesis, in which Sag functions as a Kras-cooperator in the lung (19), or Kras-antagonist in the skin (36). Our microarray analysis on bone marrow provided some mechanistic insights (Figures 5D,E). Specifically, deletion of *Sag* in bone marrow cells causes decreased induction of Mpo and Elane, two cytoprotective proteins specifically expressed in neutrophils in response to LPS (Figure 5F). Mpo, a major peroxidase expressed mainly in neutrophils to produce cytotoxic and microbicidal reactive oxidants during inflammatory response (37, 38), plays a protective role during LPS-induced endotoxemia (17). Moreover, Mpo-deficient mice exhibited elevated levels of blood cytokines and chemokines, and increased mortality in response to LPS (17). Consistent with this study, we found that the induction of Mpo in response to LPS was decreased in Sag-deficient bone marrow cells (Figure 5F). It is known that neutrophils are the major source of Mpo after LPS challenge *in vivo* (17). Thus, our finding suggests that the decreased induction of Mpo from KO mice might be associated with the increased levels of TNFα and IL-6 in sera and significantly greater susceptibility of KO mice to LPS (Figures 1B–D). In addition, the decreased induction of Elane, also known as neutrophils elastase, which is a serine protease expressed by neutrophils to degrade bacteria during inflammation (18), was found in Sag-deficient bone marrow in response to LPS. This may reduce host defense against invading pathogens, leading to the increased LPS-induced mortality in KO mice with Sag deficient myeloid cells. Future studies will be directed to further dissect the underlying mechanism by which Sag positively regulates the expression of Mpo and Elane in neutrophils.

In summary, our findings demonstrate that Sag protein differentially regulates inflammatory responses of myeloid cell subsets. In macrophages, disruption of Sag reduces the release of inflammatory cytokines by preventing the degradation of IκBα, which blocks translocation of NF-κB to the nucleus. In neutrophils, disruption of Sag has no effects on nuclear translocation of NF-κB, but may impair the induction of Mpo and Elane in response to LPS stimulation, which increases the release of inflammatory cytokines and thus likely contributing to the increased mortality *in vivo* in response to LPS.

## Author Contributions

XX and NM designed and performed the experiments, analyzed and interpreted the data, and drafted the manuscript. HuL, MT, and HF performed the experiments. HaL analyzed the data. PR and YS designed the work, analyzed and interpreted the data, and revised the manuscript. All authors reviewed the manuscript.

### Conflict of Interest Statement

The authors declare that the research was conducted in the absence of any commercial or financial relationships that could be construed as a potential conflict of interest.
