# Supplementary material for: SAG/RBX2 E3 Ubiquitin Ligase Differentially Regulates Inflammatory Responses of Myeloid Cell Subsets
Source: Front Immunol. 2018 Dec 6;9:2882. doi: 10.3389/fimmu.2018.02882 (PMC6291737; doi:10.3389/fimmu.2018.02882)

Supplemental Information

SAG/RBX2 E3 Ubiquitin Ligase Differentially Regulates

Inflammatory Responses of Myeloid Cell Subsets

Xiufang Xiong, Nathan D. Mathewson, Hua Li, Mingjia Tan, Hideaki Fujiwara, Haomin Li, Pavan Reddy, and Yi Sun

MATERIALS AND METHODS

Dendritic cell preparation

Bone marrow cells were flushed from the femurs of 8- to 12-week- old female C57BL/6 mice and cultured in 150 x 15-mm CytoOne petri dishes (USA Scientific, Ocala, FL) at $1x10^7$ in 20ml of RPMI supplemented with 10% FCS, 4mM L-glutamine, 10 U/ml penicillin, 100 μg/ml streptomycin, 0.5 mM 2-ME, 20ng/ml GM-CSF. On day 4, 20ml of fresh complete RPMI containing 20 ng/ml GM-CSF was added to each culture. After 7 days of culture, the loosely adherent cells were harvested and purified using anti-CD11c magnetic microbeads (Miltenyi Biotec Ltd., Auburn, CA) and the autoMACS (Miltenyi Biotec).

FIGURE LEGENDS

Supplemental Figure 1. Representative phenotype data.

(A) Total cells present in the spleen (left), peripheral lymph node (PLN, middle), and bone marrow (BM, right) in naïve WT (n=8) and KO (n=8) mice. Percent expression of indicated marker in the (B) spleen, (C) bone marrow, and (D) pooled peripheral

lymph nodes. * $p<0.05$.

Supplemental Figure 2. Flow cytometry gating scheme for cell analysis.

Cells isolated were gated on $CD11b^+$ and $F4.80^+$ (red box highlight) to examine macrophages in the (A) peritoneum and (B) spleen. Macrophages were analyzed for cytokine production following incubation with golgi stop protein transport inhibitor for 6 hours. (C) Neutrophils were examined in whole splenocyte populations by gating on $Gr\text{-}1^+$ and $CD11b^+$ cells (red highlight) and measuring cytokine expression.

Supplemental Figure 3. Microarray analysis results.

(A) Sag expression is significantly decreased in bone marrow cells from LysM-Cre^+/$Sag^{fl/fl}$ mice. Bone marrow cells were flushed from LysM-Cre^-/$Sag^{fl/fl}$ wild type (WT) and LysM-Cre^+/$Sag^{fl/fl}$ knockout (KO) mice, and then subjected to mRNA extraction, followed by RT-PCR. (B-C) Gene ontology (GO) analysis of 1141 genes whose expression was specifically altered in response to LPS in bone marrows from KO mice (B). #: the set of genes responsive to LPS. The genes responsive to LPS in (B) were listed (C).

Supplemental Figure 4. No significant difference in cytokine production from LPS-stimulated WT and KO dendritic cells that did not express LysM enzyme.

(A) TNFα and (B) IL-6 cytokines released and (C-D) corresponding mRNA expression by bone marrow derived dendritic cells (BMDCs) stimulated with LPS (100ng/ml) for 6 hours (n=5 for each genotype).

Supplemental Figure 1

Xiong et. al.

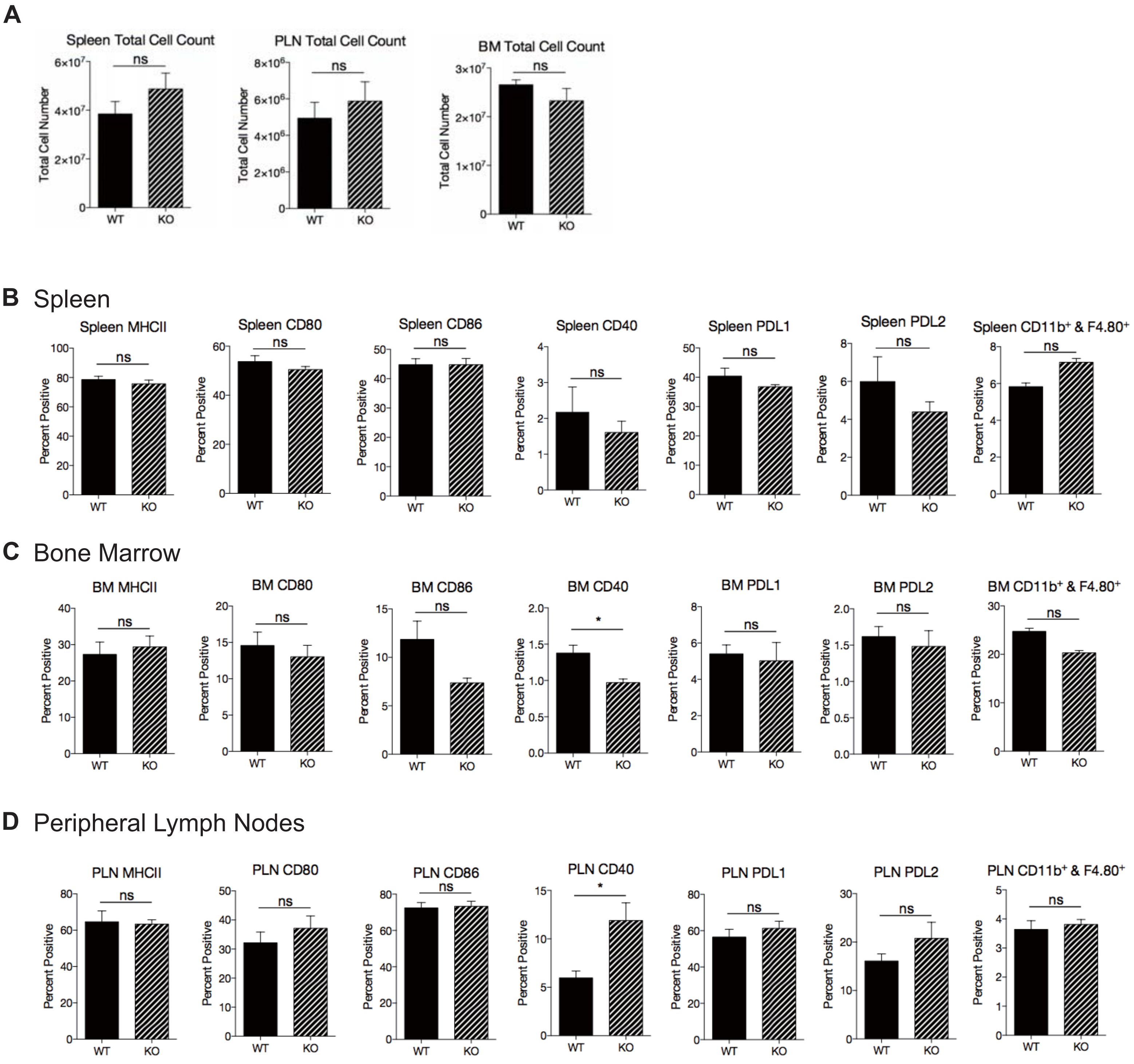

Supplemental Figure 2

Xiong et. al.

A Peritoneal macrophage gating - Unstimulated

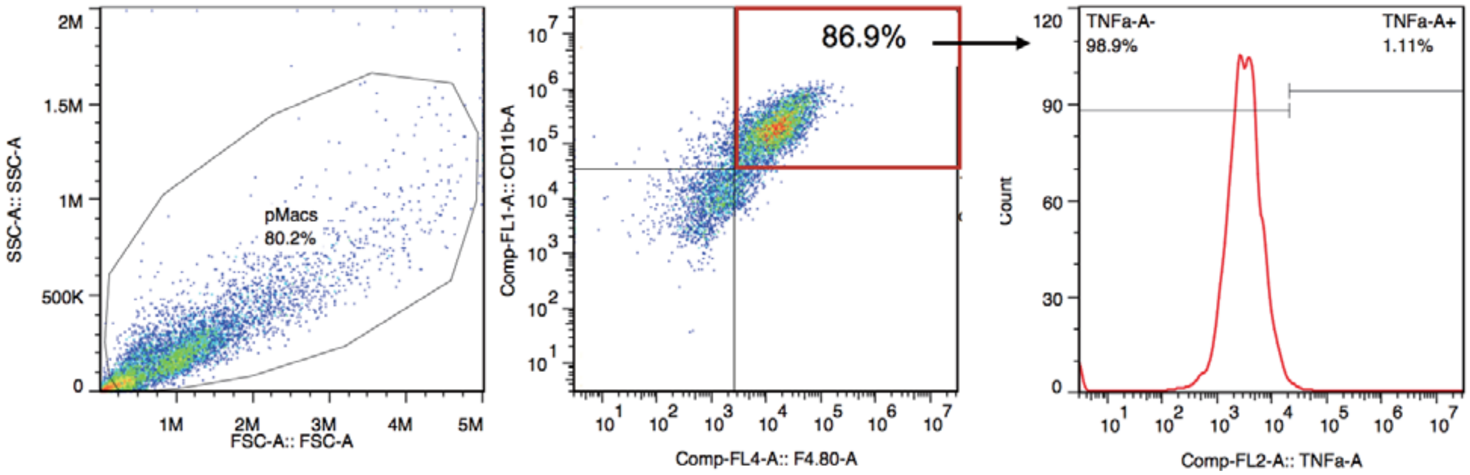

B Splenic macrophages gating - Unstimulated

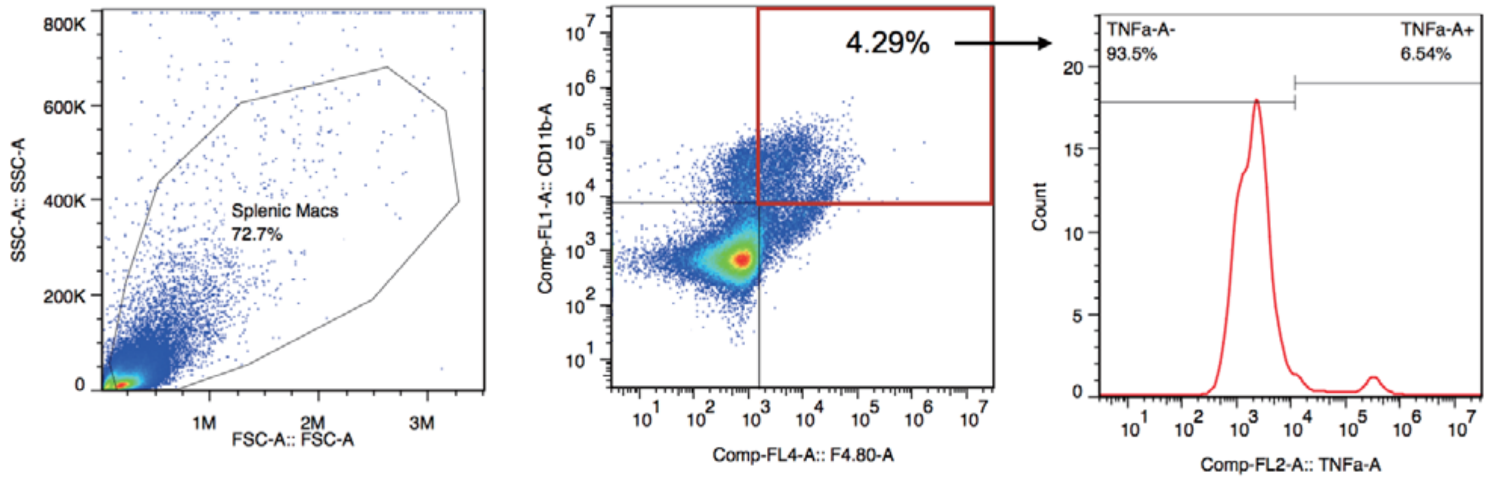

C Splenic neutrophil gating

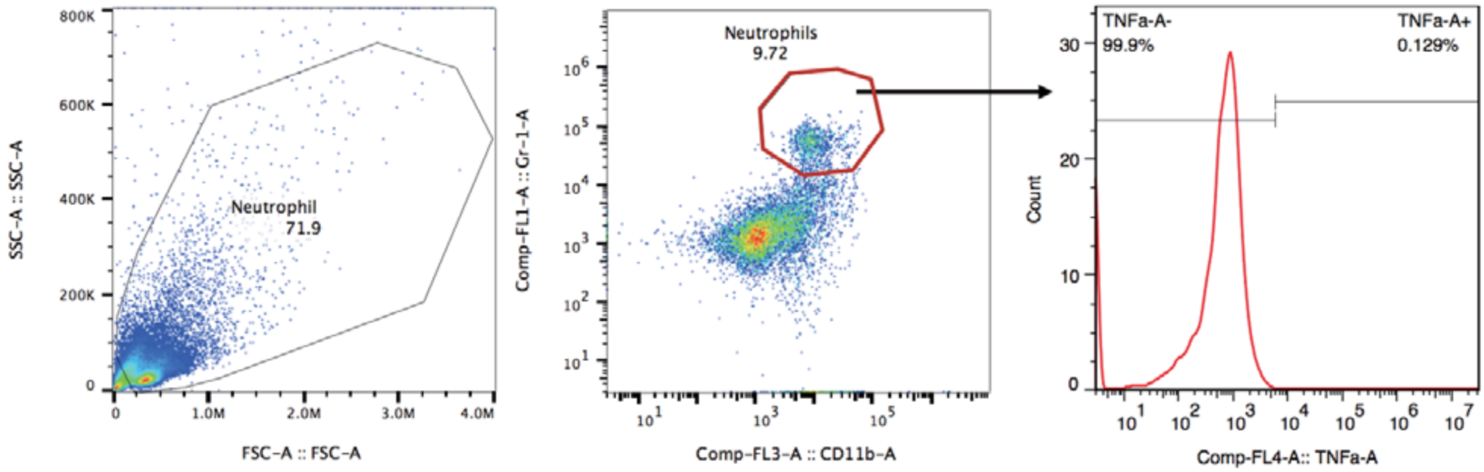

Supplemental Figure 3 Xiong et. al.

A

	LPS 0h				LPS 6h			
Sag	WT	WT	KO	KO	WT	WT	KO	KO

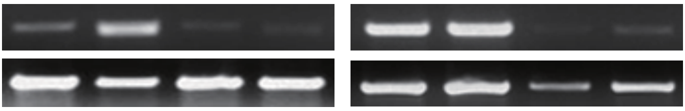

B

GO analysis (1141 genes)

Sublist	Category	Term	RT	Genes	Count	%	P-Value	Benjamini
	GOTERM_BP_DIRECT	defense response to Gram-positive bacterium	RT		18	2.0	1.6E-7	4.7E-4
	GOTERM_BP_DIRECT	oxidation-reduction process	RT		55	6.1	1.4E-6	2.0E-3
	GOTERM_BP_DIRECT	phagocytosis	RT		11	1.2	3.7E-5	3.4E-2
	GOTERM_BP_DIRECT	negative regulation of B cell proliferation	RT		7	0.8	5.2E-5	3.6E-2
	GOTERM_BP_DIRECT	carbohydrate metabolic process	RT		22	2.4	9.5E-5	5.3E-2
	GOTERM_BP_DIRECT	negative regulation of growth of symbiont in host	RT		7	0.8	1.0E-4	4.7E-2
	GOTERM_BP_DIRECT	B cell apoptotic process	RT		6	0.7	1.0E-4	4.2E-2
	GOTERM_BP_DIRECT	innate immune response in mucosa	RT		7	0.8	1.4E-4	4.8E-2
	GOTERM_BP_DIRECT	receptor internalization	RT		9	1.0	1.7E-4	5.1E-2
	GOTERM_BP_DIRECT	fatty acid metabolic process	RT		18	2.0	1.9E-4	5.4E-2
	GOTERM_BP_DIRECT	antibacterial humoral response	RT		8	0.9	2.9E-4	7.3E-2
	GOTERM_BP_DIRECT	metabolic process	RT		36	4.0	3.0E-4	6.9E-2
	GOTERM_BP_DIRECT	positive regulation of phagocytosis, engulfment	RT		5	0.6	6.9E-4	1.4E-1
	GOTERM_BP_DIRECT	cell cycle	RT		41	4.5	2.1E-3	3.5E-1
	GOTERM_BP_DIRECT	cytokinesis	RT		7	0.8	3.4E-3	4.8E-1
	GOTERM_BP_DIRECT	B cell homeostasis	RT		6	0.7	3.4E-3	4.6E-1
	GOTERM_BP_DIRECT	positive regulation of angiogenesis	RT		13	1.4	3.6E-3	4.5E-1
	GOTERM_BP_DIRECT	positive regulation of T cell proliferation	RT		9	1.0	4.1E-3	4.8E-1
	GOTERM_BP_DIRECT	mitotic nuclear division	RT		22	2.4	4.4E-3	4.9E-1
	GOTERM_BP_DIRECT	aging	RT		16	1.8	4.5E-3	4.7E-1
	GOTERM_BP_DIRECT	positive regulation of autophagosome assembly	RT		4	0.4	4.6E-3	4.7E-1
	GOTERM_BP_DIRECT	regulation of interleukin-2 biosynthetic process	RT		3	0.3	4.8E-3	4.6E-1
	GOTERM_BP_DIRECT	cell division	RT		27	3.0	5.3E-3	4.8E-1
#	GOTERM_BP_DIRECT	response to lipopolysaccharide	RT		17	1.9	6.3E-3	5.3E-1
	GOTERM_BP_DIRECT	pre-B cell differentiation	RT		4	0.4	6.4E-3	5.2E-1
	GOTERM_BP_DIRECT	lipid metabolic process	RT		31	3.4	6.9E-3	5.3E-1
	GOTERM_BP_DIRECT	hemopoiesis	RT		10	1.1	7.8E-3	5.6E-1
	GOTERM_BP_DIRECT	positive regulation of erythrocyte differentiation	RT		5	0.6	9.1E-3	6.1E-1
	GOTERM_BP_DIRECT	cellular response to mercury ion	RT		3	0.3	9.2E-3	6.0E-1
	GOTERM_BP_DIRECT	negative regulation of interleukin-8 biosynthetic process	RT		3	0.3	9.2E-3	6.0E-1
	GOTERM_BP_DIRECT	phospholipid biosynthetic process	RT		8	0.9	9.4E-3	5.9E-1
	GOTERM_BP_DIRECT	positive regulation of apoptotic process	RT		24	2.7	9.6E-3	5.9E-1
	GOTERM_BP_DIRECT	movement of cell or subcellular component	RT		5	0.6	1.1E-2	6.2E-1
	GOTERM_BP_DIRECT	positive regulation of phagocytosis	RT		7	0.8	1.2E-2	6.6E-1
	GOTERM_BP_DIRECT	sphingolipid metabolic process	RT		6	0.7	1.4E-2	7.0E-1

C

OFFICIAL_GENE_SYMBOL	GENE NAME	Related Genes	Species
Fasl	Fas ligand (TNF superfamily, member 6)(Fasl)	RG	Mus musculus
Adm	adrenomedullin(Adm)	RG	Mus musculus
Ace	angiotensin I converting enzyme (peptidyl-dipeptidase A) 1(Ace)	RG	Mus musculus
Cxcl1	chemokine (C-X-C motif) ligand 1(Cxcl1)	RG	Mus musculus
Cxcl13	chemokine (C-X-C motif) ligand 13(Cxcl13)	RG	Mus musculus
C5ar1	complement component 5a receptor 1(C5ar1)	RG	Mus musculus
Elane	elastase, neutrophil expressed(Elane)	RG	Mus musculus
Hmgb2	high mobility group box 2(Hmgb2)	RG	Mus musculus
Lbp	lipopolysaccharide binding protein(Lbp)	RG	Mus musculus
Mgst2	microsomal glutathione S-transferase 2(Mgst2)	RG	Mus musculus
Maob	monoamine oxidase B(Maob)	RG	Mus musculus
Mpo	myeloperoxidase(Mpo)	RG	Mus musculus
Pck2	phosphoenolpyruvate carboxykinase 2 (mitochondrial)(Pck2)	RG	Mus musculus
Plcg2	phospholipase C, gamma 2(Plcg2)	RG	Mus musculus
Snca	synuclein, alpha(Snca)	RG	Mus musculus
Tnfrsf21	tumor necrosis factor receptor superfamily, member 21(Tnfrsf21)	RG	Mus musculus
Tnfrsf4	tumor necrosis factor receptor superfamily, member 4(Tnfrsf4)	RG	Mus musculus

Supplemental Figure 4

Xiong et. al.

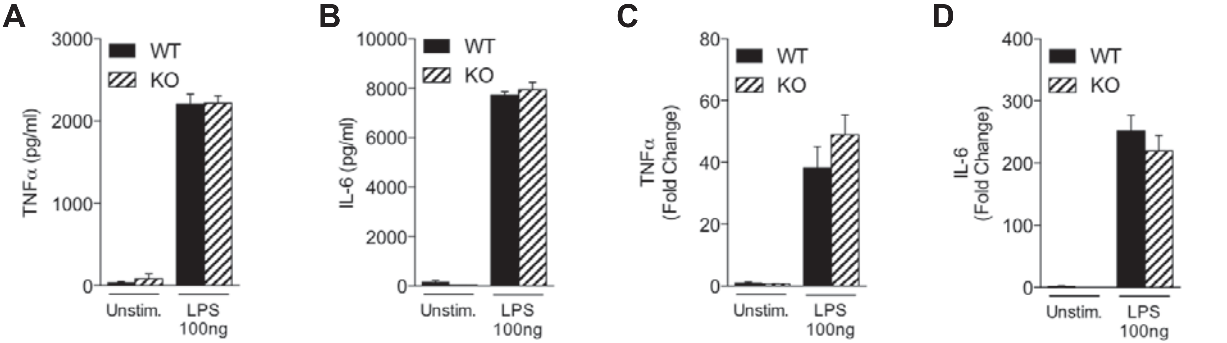

Supplement: Supplementary file 1 [file Data_Sheet_1.PDF]
